# A mathematical analysis of math anxiety dynamics using a classical SAS model: Bridging epidemic theory and pedagogical implications

**DOI:** 10.1371/journal.pone.0351626

**Published:** 2026-07-01

**Authors:** Shobha Islam, Dulali Iqbal, Mili Saha, Goutam Saha

**Affiliations:** 1 Department of Mathematical and Physical Sciences, East West University, Dhaka, Bangladesh; 2 Department of Mathematics and Statistics, Bangladesh University of Business and Technology, Dhaka, Bangladesh; 3 Department of Mathematics and Natural Sciences, BRAC University, Dhaka, Bangladesh; 4 Department of English, Jagannath University, Dhaka, Bangladesh; 5 Miyan Research Institute, International University of Business Agriculture and Technology, Dhaka, Bangladesh; 6 Department of Mathematics, University of Dhaka, Dhaka, Bangladesh; 7 Department of Mathematics, International University of Business Agriculture and Technology, Dhaka, Bangladesh; University of Louisiana at Lafayette, UNITED STATES OF AMERICA

## Abstract

Students’ cognitive function and participation in mathematical problems are adversely affected by mathematics anxiety. This research develops and evaluates a dynamic mathematical model (SAS: Susceptible-Anxious-Susceptible) to investigate the evolution and transmission of math anxiety within students over time. The model divides students into two subgroups based on presence and absence of math anxiety. Using epidemiological modeling concepts, we derive the basic reproduction number R₀, which determines the conditions for persistence (R₀  >  1) or elimination (R₀  <  1) of anxiety. Our analysis proves that the anxiety-free equilibrium is both locally and globally asymptotically stable when R₀  <  1, while the anxiety-prevailing equilibrium is stable when R₀  >  1, with the system undergoing a transcritical bifurcation at R₀  =  1. Sensitivity analysis using Partial Rank Correlation Coefficient (PRCC) reveals that the university entrance rate (π) and social transmission rate (β) positively impact R₀, while recovery rate (α) and exit rate (μ) negatively influence it. Notably, π and μ are identified as the most influential parameters. Numerical simulations demonstrate that the population of students with mathematics anxiety is highly sensitive to changes in β and μ, while remaining relatively stable with fluctuations in π and α. These findings provide a quantitative framework for developing effective interventions, suggesting that reducing social transmission of anxiety and enhancing recovery through supportive mechanisms can significantly curb math anxiety prevalence in educational settings.

## Introduction

Mathematics anxiety is a psychological condition characterized by feelings of tension, apprehension, and fear when engaging with mathematical tasks or problem-solving. These emotional responses can significantly impair an individual’s ability to perform mathematical operations and, in turn, negatively affect their educational and professional outcomes. Various factors contribute to the development of mathematics anxiety, including low self-confidence in mathematical abilities, negative past experiences with mathematics, and internalized beliefs about being inherently “not good at math.”

Emerging research indicates a potential relationship between mathematics anxiety and gender. Studies consistently report that, on average, females experience higher levels of mathematics anxiety than males [1]. This disparity may be influenced by sociocultural expectations, pedagogical practices, and cognitive differences in how males and females approach mathematical tasks. Elevated anxiety levels among female students have been linked to lower mathematical performance compared to their male counterparts [1].

A well-documented inverse relationship exists between mathematics anxiety and academic performance. Individuals with higher levels of math anxiety generally achieve lower mathematics grades compared to those with lower anxiety levels [2]. This may be attributed to anxiety’s disruptive effects on focus, logical reasoning, and problem-solving abilities—skills critical for success in mathematics. Furthermore, individuals with heightened anxiety often exhibit avoidance behaviors or procrastination regarding mathematical tasks, further hindering their performance. The impact of mathematics anxiety extends beyond mathematics-specific outcomes. Research suggests that students with high mathematics anxiety tend to have lower overall academic achievement, reduced participation in classroom and extracurricular activities, and decreased likelihood of pursuing higher education or careers in STEM-related fields [3,4].

Math anxiety (MA) refers to the tense, uneasy, or frightening feelings that impair an individual’s capacity to perform mathematical tasks. It can emerge in everyday activities such as budgeting or tip calculation as well as in academic contexts. Studies show that over 17% of Americans suffer from severe math anxiety, which negatively affects performance and often leads to avoidance of math-related situations [5]. Research indicates that MA typically first appears in elementary school, intensifies during adolescence, and is strongly correlated with academic achievement [6–9]. These longitudinal findings highlight gendered patterns and developmental cycles in the trajectory of MA. Several mechanisms explain how MA disrupts performance. Devine et al. [10] measured math anxiety, while Núñez-Peña et al. [11] emphasized cognitive interference. Spatial anxiety has been proposed as a mediator of sex differences [12], and neuroscientific evidence shows that males and females follow distinct pathways in linking self-efficacy and spatial ability [13]. Ashcraft and Moore [14] concluded that working memory disturbance and avoidance are the two central mechanisms underlying MA. Measurement tools have been refined to study MA across populations. The Abbreviated Math Anxiety Scale (AMAS) was validated by Primi et al. [15] and later extended to middle school students by Cohen and Limbers [16], confirming its two-factor structure and gender invariance. Psychometric validation ensures comparability across cultures and age groups, thereby supporting survey-experimental and intervention research. Cognitive-experimental studies also reveal broader consequences: Maloney and Retanal [17] showed that MA lowers need for cognition and reflective reasoning beyond math contexts, while Cohen et al. [18] found that math-related words carry stronger negative valence, especially for women.

Mathematics anxiety is shaped by social experiences, stereotypes, and cultural contexts. Henschel and Roick [19] differentiated between cognitive and affective math anxiety, while Mizala et al. [20] showed that pre-service teachers’ MA affects their capacity of developing inclusive learning environments. Similarly, Maloney et al. [21] linked MA to stereotype threat and negative consequences. Gender and cultural differences are well-documented: Xie et al. [22] and Sorvo et al. [8] showed that test anxiety and self-esteem mediate MA differently in Chinese and Finnish samples, while Casanova et al. [23] found that Latina and Black girls are disproportionately affected by math anxiety. Importantly, Flessati and Jamieson [24] showed that sex differences in MA may arise due to inaccurate responses. The relationship between MA and achievement is reciprocal. Carey et al. [25] demonstrated causal links between low achievement and high anxiety, while Hembree’s [5] meta-analysis consistently confirmed negative associations. Devine et al. [6] updated effect estimates, and recent modeling work illustrated how MA spreads across teacher and peer networks, reinforcing systemic barriers in mathematics learning [26].

Resilience-based perspectives highlight how self-concept and coping strategies influence outcomes. Latterell and Wilson [27] reported that students’ mathematical autobiographical narratives can help teachers improve their future mathematics instruction. Morán-Soto and González-Peña [28] emphasized self-efficacy as a protective factor, particularly in engineering education. Intervention research further shows that growth-mindset practices, structured pedagogy, and collaborative teamwork reduce MA [29]. An SIR-type mathematical model was also developed by Kaymakamzade and Cumhur [30] to describe the dynamics of math anxiety in college students. In order to depict performance levels and anxiety susceptibility, the model separates the population into three groups: weak, average, and above-average students. The transmission rate of anxiety is represented by parameters such as β₁ and β₂, whilst behavioral change influenced by instructional quality is indicated by γ and τ. Their simulations demonstrate how instructional techniques that boost students’ self-esteem and encourage constructive involvement might lessen anxiety. This model demonstrates how anxiety can be viewed as a “infectious” cognitive-emotional condition that is spread through social and intellectual contact, bridging the gap between affective science and applied mathematics.

Recent research shows very clearly that school-based mental-health and resilience programs can make a real difference in how children and adolescents manage their emotions, cope with stress, and perform academically. One of the earlier studies in this area, by Lee et al. [31], found that school-delivered cognitive behavioral therapy (CBT) reduced anxiety levels in children, with improvements lasting for up to two years. Although the effects lessened by the three-year follow up, the study still demonstrates that CBT works well in a school environment. This is especially important because math anxiety is essentially a form of performance anxiety, and CBT is one of the most effective treatments for these types of fears. Bringing CBT strategies such as challenging negative thoughts, slowly increasing exposure to difficult problems, and learning coping skills into math classrooms could therefore help reduce anxiety in the same way.

More recent studies have explored different types of interventions. Sun et al. [32] showed that even a short mindfulness practice (only eight minutes a day for five days) lowered test anxiety and boosted exam performance in high-school students. Since math anxiety often comes with strong physical reactions (like a racing heart or tense muscles) and emotional stress during math tasks, mindfulness can help students calm their bodies and minds before and during lessons. The improvement in performance is also significant, because math anxiety is known to interfere with working memory. When students feel calmer, their ability to think clearly during math tasks improves.

Liu et al. [33] provide another valuable insight through their study that group-based CBT sessions helped primary school students build resilience and experience more positive academic emotions. This matters because math anxiety is closely linked to feelings of fear, shame, frustration, and low confidence. Teaching children how to reframe negative thoughts, manage their emotions, and practice skills in a supportive group can reduce these negative emotions and interrupt the cycle of avoidance that often accompanies math anxiety. Large-scale evidence from meta-analyses by Cai et al. [34] and Phan et al. [35] also shows that resilience-building, mindfulness, and CBT programs lead to small but meaningful improvements in coping and emotional regulation. Math anxiety is, at its core, a breakdown of coping. Students feel overwhelmed, freeze during tasks, or avoid math altogether. Strengthening resilience especially through interventions embedded in the school curriculum can help students develop the persistence and emotional flexibility needed to engage with challenging math contents. Even small shifts in resilience can move a student from “avoidance mode” to “approach mode,” gradually lowering math anxiety. Studies conducted in low and middle-income countries further show that these interventions remain effective even in schools with limited mental-health resources or large class sizes [36]. This is reassuring, because math anxiety often disproportionately affects students from disadvantaged backgrounds. If simple, scalable programs such as short mindfulness activities, group counselling, or teacher-led resilience lessons work in resource-constrained institutions, they can also be adapted widely to address math anxiety in many different educational contexts.

At the classroom level, teacher-led interventions play an important role in reducing math anxiety. When teachers provide emotional support, show encouragement, and display enthusiasm, they help create a positive learning environment that lowers students’ feelings of fear or stress during mathematics lessons [37]. Social strategies also contribute to reducing anxiety. Peer modelling and cooperative learning allow students to learn from one another, which increases confidence and reduces stress through vicarious experiences [38]. In addition, instructional approaches such as conceptual tutoring and metacognitive strategy training help students better understand mathematical ideas and manage problem-solving steps. This improved sense of mastery reduces confusion and decreases math anxiety [39]. Finally, new research into digital mental-health tools shows promising results for school-based e-health interventions [40]. This aligns with emerging math anxiety research, which highlights the usefulness of digital supports such as guided exposure, self-paced math practice with supportive feedback, and apps that offer breathing or mindfulness exercises during lessons. These tools provide flexible, accessible ways to support students and could become an important part of future math anxiety interventions.

These evidences altogether demonstrate that despite equal ability, MA undermines performance and persistence in mathematics, particularly among women and underrepresented groups. Findings converge across psychometric, experimental, longitudinal, qualitative, and intervention approaches, strengthening causal claims. Although the reviewed studies comprehensively address measurement, cognition, gender, culture, and intervention, very few research have been conducted in incorporating the math anxiety data to formulate a mathematical model which does not include the detailed analysis on how the model changes with the change in parameter and how to control the anxiety level based on the analysis. Our aim is to establish a model demonstrating math anxiety and analyze its dynamical behavior.

The remaining part of this paper is organized as follows:

- SAS model describing math anxiety dynamics is formulated.
- Some fundamental properties of the model are derived.
- Equilibrium points are calculated along with stability analysis.
- Addresses the bifurcation analysis of the model.
- Sensitivity index of basic reproduction number is presented.
- Numerical simulations of the model are performed.
- Outlines some strategies for controlling mathematics anxiety.
- Concluding remarks are presented.

## Mathematical model

In this section, we formulate a model where S is the susceptible class of students who do not have mathematics anxiety and A is the class of students who have mathematics anxiety. Here, some students can recover math anxiety temporarily and transfer into the susceptible class.


$$\begin{array}{c} \;\begin{array}{c} \frac{dS}{dt}=\pi -\beta SA+\alpha A-\mu S\\ \frac{dA}{dt}=\beta SA-\;\alpha A-\;\mu A \end{array}\} \end{array}$$ (1)

with initial conditions


$$\begin{array}{c} S(\text{0})>\text{0},A(\text{0})\geq \text{0}. \end{array}$$ (2)

where,

S: Susceptible students (do not have math anxiety yet, but may develop math anxiety)

A: Students with math anxiety

and total number of students, P(t)=S(t)+A(t).

Also,

Π: The University entrance rate. The constant rate at which new students enter the university and join the susceptible class (S) per unit time. This parameter represents the influx of students who are initially free of math anxiety.

*β*: The transfer rate of susceptible students to students with math anxiety due to social interaction.

*α*: The transfer rate from mathematics anxiety to susceptible class due to social interactions or personal decision on reducing anxiety. Research on coping strategies, counseling, and interventions shows students can reduce their anxiety over time [31–34]. This directly connects to the part of our model where anxious students can recover and return to the “susceptible” group (rate α).

*μ*: The rate of graduation, dismissal or drop-out. Students graduating or dropping out is a natural part of school populations. This motivates the “removal rate” (μ) in our model, which keeps the population realistic.

In this study, the total student population is divided into two mutually exclusive compartments: the susceptible group *S*, consisting of students who do not currently experience mathematics anxiety, and the anxious group *A*, consisting of students who exhibit anxiety. Students enter the system at an admission rate *Π* and join the susceptible class. Through social interaction with anxious peers, susceptible students may acquire math anxiety and transition to the anxious class at a transmission rate *β*. This term is modeled using a bilinear incidence function *βSA*, which is widely used in models of affective and behavioral contagion when the likelihood of transmission increases with both the number of susceptible individuals and the number of exposed individuals. The use of this bilinear contact term is grounded in empirical evidence from educational psychology showing that anxiety, negative emotions, and maladaptive beliefs can spread through peer interactions, classroom climate, stereotype activation, and teacher–student affective exchange. Several studies have demonstrated that students’ anxiety levels are influenced by the emotional states of teachers [20], peers [21], and classroom networks [26]. Also, research on affective transmission further indicates that negative emotions, avoidance behaviors, and stereotype-driven discourse can propagate through social channels, shaping other students’ anxiety responses [18,25]. Similar bilinear incidence structures have been adopted in prior mathematical models of academic anxiety [30], supporting its applicability to the present context.

Moreover, students in the anxious class may recover from anxiety and return to the susceptible class at rate *α*, representing personal coping, support interventions, or counseling. All students exit the system through graduation, dismissal, or dropout at a per-capita rate μ. The resulting model captures the dynamic interplay between social influence, recovery, and academic turnover in shaping math anxiety within a student population.

**Fig 1:**
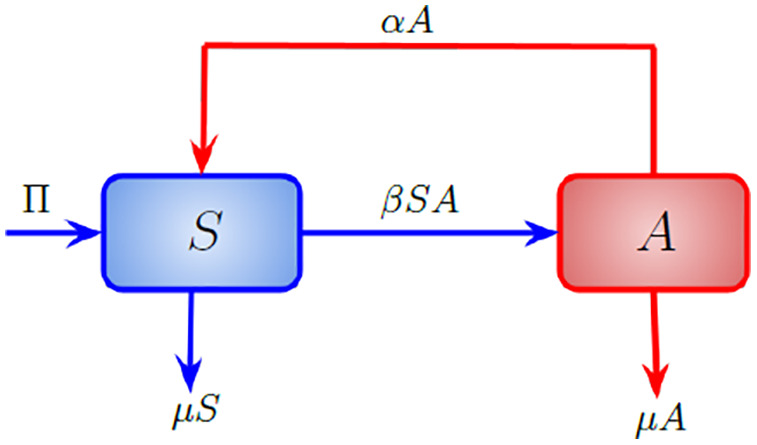
Flow diagram of the model.

The flow diagram of the SAS model is presented in Fig 1, and the model parameters with their descriptions are listed in Table 1.

**Table 1:** Parameters and their descriptions of SAS model.

Parameters	Description	Values	Units
π	University entrance rate	(0.001, 0.009)	Per day
β	Transmission rate from S to A class	(0.001, 0.009)	Per contact
α	Transferring rate from A to S class through consultation	(0.002, 0.006)	Per day
μ	Rate of graduation, dismissal, or drop-out	(0.0002, 0.0008)	Per day

### Fundamental properties of the deterministic model

#### Existence of unique solution.

**Theorem 1:** The model system (1) together with the initial conditions (2) has a unique solution.

Proof: Let us assume that $y_{\text{1}}=S,{\;y}_{\text{2}}=A$ and $\vec{y}={\left({\;y}_{\text{1}},\;{\;y}_{\text{2}}\right)}^{T}$.

Also let us consider,


$$\begin{array}{c} \;\begin{array}{c} {\;g}_{\text{1}}=\;\pi -\beta {\;y}_{\text{1}}{\;y}_{\text{2}}+{\alpha y}_{\text{2}}-\mu y_{\text{1}},\\ {\;g}_{\text{2}}=\beta {\;y}_{\text{1}}{\;y}_{\text{2}}-{\alpha y}_{\text{2}}-\mu {\;y}_{\text{2}} \end{array}\} \end{array}$$

where $\vec{g}={\left({\;g}_{\text{1}},\;{\;g}_{\text{2}}\right)}^{T}.$

Then the system (1) can be written as


$$\begin{array}{c} \frac{d\vec{y}}{dt}=\vec{g}\left(\vec{y}\right), \end{array}$$

with initial conditions,


$$\begin{array}{c} \vec{y}(\text{0})={\left({\;y}_{\text{1}}(\text{0}),\;{\;y}_{\text{2}}(\text{0})\right)}^{T}. \end{array}$$

Clearly, $g_{\text{1}}$ and $g_{\text{2}}$ are continuous on $R^{\text{2}}$ and also is $\vec{g}$. In addition, the partial derivatives


$$\begin{array}{c} \frac{\partial g_{i}}{\partial {\;y}_{j}}\;(\text{1}\leq i,j\leq \text{2}) \end{array}$$

exists and are continuous on $R^{\text{2}}$. Therefore, by existence and uniqueness theorem, we conclude that there exists a unique solution to the model systems (1).

#### Non-negativity of the model solution.

**Theorem 2:** All the solutions of the math anxiety model (1) are non-negative given that the initial conditions (2) are non-negative.

Proof: The first differential equation of the system (1) can be written as


$$\begin{array}{c} \frac{dS}{dt}=\pi -\beta SA+\alpha A-\mu S \end{array}$$

$$\begin{array}{c} \Rightarrow \frac{dS}{dt}+\beta SA+\mu S=\pi +\alpha A \end{array}$$

$$\begin{array}{c} \Rightarrow \frac{dS}{dt}+(\beta A+\mu )S=\pi +\alpha A. \end{array}$$

Solving the above differential equation, we get,


$$\begin{array}{c} S(t)e^{\int_{\text{0}}^{t}(\beta A+\mu )\;ds}-S(\text{0})=\int_{\text{0}}^{t}{\{(\pi +\alpha A)e}^{\int_{\text{0}}^{t}(\beta A+\mu )ds}\}dt \end{array}$$

$$\begin{array}{c} \Rightarrow S(t)=e^{-\int_{\text{0}}^{t}(\beta A+\mu )ds}[S(\text{0})+\int_{\text{0}}^{t}{\{(\pi +\alpha A)e}^{\int_{\text{0}}^{t}(\beta A+\mu )ds}\}dt]\geq \text{0}, \end{array}$$

since S(0)≥0.

Again, from the second equation of the system (1), we get,


$$\begin{array}{c} \frac{dA}{dt}=\beta SA-\;\alpha A-\;\mu A \end{array}$$

$$\begin{array}{c} \Rightarrow \frac{dA}{dt}\geq -(\mu +\alpha )A \end{array}$$

$$\begin{array}{c} \Rightarrow \frac{dA}{A}\geq -(\mu +\alpha )dt. \end{array}$$

Solving the inequality, we have,


$$\begin{array}{c} A(t)\geq A(\text{0})e^{-(\mu +\alpha )t}\geq \text{0}, \end{array}$$

since A(0)≥0.

#### Boundedness of the model solution.

**Theorem 3:** The solution of the model system (1) is bounded in the compact region


$$\begin{array}{c} \Omega =\left\{(S,\;A)\in \overset{\text{2}}{\underset{+}{R}}:\text{0}<P\leq \frac{\pi }{\mu }\right\}. \end{array}$$

Proof: Let, $(S,\;A)\in \overset{\text{2}}{\underset{+}{R}}$ be any solution of the system of equations (1) with non-negative initial conditions. We know,


$$\begin{array}{c} P(t)=S(t)+A(t) \end{array}$$

$$\begin{array}{c} ⟹\frac{dP}{dt}=\frac{dS}{dt}+\frac{dA}{dt} \end{array}$$

$$\begin{array}{c} \Rightarrow \frac{dP}{dt}=\pi -\beta SA+\alpha A-\mu S\beta SA-\;\alpha A-\;\mu A=\pi -\mu S-\;\mu A \end{array}$$

$$\begin{array}{c} ⟹\frac{dP}{dt}=\pi -\mu (S+A)=\pi -\mu P \end{array}$$

$$\begin{array}{c} ⟹\frac{dP}{dt}+\mu P=\pi . \end{array}$$

Now, applying the integrating factor method,


$$\begin{array}{c} P(t)e^{\mu t}-P(\text{0})=\int_{\text{0}}^{t}{\{\pi e}^{\int_{\text{0}}^{t}\mu s\;ds}\}dt \end{array}$$

$$\begin{array}{c} \Rightarrow \;P(t)e^{\mu t}=P(\text{0})+\frac{\pi }{\mu }\left(e^{\mu t}-\text{1}\right) \end{array}$$

$$\begin{array}{c} ∴\;P(t)=P(\text{0})e^{-\mu t}+\frac{\pi }{\mu }\left(\text{1}-e^{-\mu t}\right)=\frac{\pi }{\mu }+e^{-\mu t}\left(P(\text{0})-\frac{\pi }{\mu }\right). \end{array}$$

When


$$\begin{array}{c} P>\frac{\pi }{\mu },\;\frac{dP}{dt}<\text{0}. \end{array}$$

Therefore,


$$\begin{array}{c} \frac{dP}{dt}\;\text{is}\;\text{bounded}\;\text{by}\;\pi -\mu P. \end{array}$$

Now,


$$\begin{array}{c} P(t)\leq P(\text{0})e^{-\mu t}+\frac{\pi }{\mu }\left(\text{1}-e^{-\mu t}\right). \end{array}$$

In particular,


$$\begin{array}{c} P(t)\leq \frac{\pi }{\mu }\text{ if }P(\text{0})\leq \frac{\pi }{\mu }\;. \end{array}$$

Hence, P(t) is bounded above by $\frac{\pi }{\mu }$. Hence the population S(t),  A(t) are bounded above by $\frac{\pi }{\mu }$.

### Analysis of the deterministic model

#### Anxiety free equilibrium.

Let, $E^{\text{0}}=(S^{\text{0}},{\;A}^{\text{0}})$ be the anxiety free equilibrium. So, $A^{\text{0}}=\text{0}$.

Now, solving the system


$$\begin{array}{c} \frac{dS}{dt}=\frac{dA}{dt}=\text{0}, \end{array}$$

We get,


$$\begin{array}{c} S^{\text{0}}=\frac{\pi }{\mu },\;{\;A}^{\text{0}}=\text{0}. \end{array}$$

Hence, the anxiety free equilibrium is


$$\begin{array}{c} E^{\text{0}}=\left(\frac{\pi }{\mu },\;\text{0}\right). \end{array}$$

#### Basic reproduction number.

The basic reproduction number is to be found by using next generation method. Here, the math anxious compartment is


$$\begin{array}{c} \frac{dA}{dt}=\beta SA-\;\alpha A-\;\mu A. \end{array}$$

Then,


$$\begin{array}{c} f=\;\beta SA\;and\;\nu =(\alpha +\mu )A. \end{array}$$

Now, F (for new infection terms) and V (for transition terms) are obtained by taking partial derivatives with respect to A as follows:


$$\begin{array}{c} F=\beta S\;and\;V=(\alpha +\mu ). \end{array}$$

At anxiety free equilibrium:


$$\begin{array}{c} FV^{-\text{1}}=\frac{\beta \pi }{\mu }\;.\;\frac{\text{1}}{\left(\alpha +\mu \right)}. \end{array}$$

Therefore, the basic reproduction number is


$$\begin{array}{c} R_{\text{0}}=\frac{\beta \pi }{\mu (\alpha +\mu )}. \end{array}$$

#### Local stability analysis of anxiety free equilibrium.

**Theorem 5:** The anxiety free equilibrium $E^{\text{0}}$ is locally asymptotically stable on Ω if $R_{\text{0}}<\text{1}$ and unstable otherwise.

Proof: The Jacobian matrix of the model (1) at $E^{\text{0}}$ is


$$\begin{array}{c} J\left(E^{\text{0}}\right)=[\begin{array}{cc} -\;\mu & -\frac{\beta \pi }{\mu }+\alpha \\ \text{0} & \frac{\beta \pi }{\mu }-(\mu +\alpha ) \end{array}]\;. \end{array}$$

To find the eigenvalues of the matrix $J\left(E^{\text{0}}\right)$, we solve its characteristic equation $\left|J\left(E^{\text{0}}\right)-\lambda I\right|=\text{0}$; where I is a 2×2 identity matrix.


$$\begin{array}{c} \left|J\left(E^{\text{0}}\right)-\lambda I\right|=\left|\begin{array}{cc} -\;\mu -\lambda_{\text{1}} & -\frac{\beta \pi }{\mu }+\alpha \\ \text{0} & \frac{\beta \pi }{\mu }-(\mu +\alpha )-\lambda_{\text{2}} \end{array}\right|=\text{0} \end{array}$$

$$\begin{array}{c} \Rightarrow \left(-\;\mu -\lambda_{\text{1}}\right)\left(\frac{\beta \pi }{\mu }-(\mu +\alpha )-\lambda_{\text{2}}\right)=\text{0} \end{array}$$ (3)

$$\begin{array}{c} \;\begin{array}{c} ∴\lambda_{\text{1}}=-\;\mu ,\\ \lambda_{\text{2}}=\frac{\beta \pi }{\mu }-(\mu +\alpha ). \end{array}\} \end{array}$$

Here, $\lambda_{\text{1}}<\text{0}$ and if $\lambda_{\text{2}}<\text{0}$, then


$$\begin{array}{c} \frac{\beta \pi }{\mu }-(\mu +\alpha )<\text{0} \end{array}$$

$$\begin{array}{c} ⟺\frac{\beta \pi }{\mu }<(\mu +\alpha ) \end{array}$$

$$\begin{array}{c} ⟺\frac{\beta \pi }{\mu (\alpha +\mu )}<\text{1} \end{array}$$

$$\begin{array}{c} ⟺R_{\text{0}}<\text{1}. \end{array}$$

Thus $\lambda_{\text{2}}<\text{0}$ if and only if $R_{\text{0}}<\text{1}$. It can be seen that all the eigenvalues of the characteristic polynomial (3) have negative real part if and only if $R_{\text{0}}<\text{1}$. Hence, it is proved that if $R_{\text{0}}<\text{1}$, then $E^{\text{0}}$ is locally asymptotically stable. Again, if $R_{\text{0}}>\text{1}$, then clearly $\lambda_{\text{2}}>\text{0}$. So, equation (3) has at least one positive eigenvalue which implies that $E^{\text{0}}$ is asymptotically unstable. Hence, the theorem is proved.

#### Global stability analysis of anxiety free equilibrium.

**Theorem 6:** The anxiety free equilibrium $E^{\text{0}}$ is globally asymptotically stable on Ω if $R_{\text{0}}<\text{1}$ and unstable otherwise.

Proof: Let us consider the following Lyapunov function


$$\begin{array}{c} L(S,A)=\int_{S^{\text{0}}}^{S}\left(\text{1}-\frac{S^{\text{0}}}{x}\right)dx+A. \end{array}$$

Here, L(S, A)>0 for $\left(S,A)=\;\;/\;(S^{\text{0}},\;A^{\text{0}}\right)$ and for $\left(S,\;A)=(S^{\text{0}},\;A^{\text{0}}\right)$,


$$\begin{array}{c} L(S,\;A)=\int_{S^{\text{0}}}^{S^{\text{0}}}\left(\text{1}-\frac{S^{\text{0}}}{x}\right)dx+A^{\text{0}}=\text{0}. \end{array}$$

Now, the derivative of L with respect to time is


$$\begin{array}{c} \frac{dL}{dt}=\left(\text{1}-\frac{S^{\text{0}}}{S(t)}\right)\frac{dS}{dt}+\frac{dA}{dt} \end{array}$$

$$\begin{array}{c} \Rightarrow \frac{dL}{dt}=\left(\text{1}-\frac{S^{\text{0}}}{S}\right)(\pi -\beta SA+\alpha A-\mu S)+\beta SA-\;\alpha A-\;\mu A \end{array}$$

$$\begin{array}{c} \Rightarrow \frac{dL}{dt}=\left(\text{1}-\frac{S^{\text{0}}}{S}\right)\left(\mu S^{\text{0}}-\beta SA+\alpha A-\mu S\right)+\beta SA-\;\alpha A-\;\mu A\;[{∵S}^{\text{0}}=\frac{\pi }{\mu }⟹\pi =\mu S^{\text{0}}] \end{array}$$

$$\begin{array}{c} \Rightarrow \frac{dL}{dt}=\left(\text{1}-\frac{S^{\text{0}}}{S}\right)\left(\mu {(S}^{\text{0}}-S)-\beta SA+\alpha A\right)+\beta SA-\;\alpha A-\;\mu A \end{array}$$

$$\begin{array}{c} \Rightarrow \frac{dL}{dt}=\left(\mu {(S}^{\text{0}}-S)\left(\text{1}-\frac{S^{\text{0}}}{S}\right)-\beta SA\left(\text{1}-\frac{S^{\text{0}}}{S}\right)+\alpha A\left(\text{1}-\frac{S^{\text{0}}}{S}\right)\right)+\beta SA-\;\alpha A-\;\mu A \end{array}$$

$$\begin{array}{c} \Rightarrow \frac{dL}{dt}=\left(-\mu \left(\frac{{{(S-S}^{\text{0}})}^{\text{2}}}{S}+A\right)+\beta SA\left(\frac{S^{\text{0}}}{S}\right)+\alpha A\left(-\frac{S^{\text{0}}}{S}\right)\right) \end{array}$$

$$\begin{array}{c} \Rightarrow \frac{dL}{dt}=\left(-\mu \left(\frac{{{(S-S}^{\text{0}})}^{\text{2}}}{S}+A\right)-\frac{{\alpha AS}^{\text{0}}}{S}+\beta S^{\text{0}}A\right) \end{array}$$

$$\begin{array}{c} \Rightarrow \frac{dL}{dt}=\left(-\mu \left(\frac{{{(S-S}^{\text{0}})}^{\text{2}}}{S}+A\right)-\frac{{\alpha AS}^{\text{0}}}{S}+\frac{\beta S^{\text{0}}A(\alpha +\mu )}{\left(\alpha +\mu \right)}\right) \end{array}$$

$$\begin{array}{c} \Rightarrow \frac{dL}{dt}=\left(-\mu \left(\frac{{{(S-S}^{\text{0}})}^{\text{2}}}{S}+A\right)-\frac{{\alpha AS}^{\text{0}}}{S}+R_{\text{0}}A(\alpha +\mu )\right). \end{array}$$

Therefore if $R_{\text{0}}<\text{1}$, then $\frac{dL}{dt}<\text{0}$ and $\frac{dL}{dt}=\text{0}$ if and only if $S=S^{\text{0}}$ and $A=A^{\text{0}}$. Hence by Lyapunov stability theorem, it is verified that $E^{\text{0}}$ is globally asymptotically stable on Ω if $R_{\text{0}}<\text{1}$ and unstable otherwise  [41].

#### Existence of anxiety prevailing equilibrium.

**Theorem 7:** The system of equations (1) has a unique positive anxiety prevailing equilibrium whenever $R_{\text{0}}>\text{1}$ and no positive equilibrium otherwise.

Proof: Let, $E^{*}=(S^{*},A^{*})$ be the anxiety prevailing equilibrium. Then, solving the system,


$$\begin{array}{c} \frac{dS^{*}}{dt}=\frac{dA^{*}}{dt}=\text{0}, \end{array}$$

We obtain,


$$\begin{array}{c} \;\begin{array}{c} S^{*}=\frac{\alpha +\mu }{\beta },\\ A^{*}=\frac{\pi }{\mu }-\frac{\alpha +\mu }{\beta }. \end{array}\} \end{array}$$

Again,


$$\begin{array}{c} A^{*}=\frac{\pi }{\mu }\left(\text{1}-\frac{\mu (\alpha +\mu )}{\beta \pi }\right) \end{array}$$

$$\begin{array}{c} =\frac{\pi }{\mu }\left(\text{1}-\frac{\text{1}}{\frac{\beta \pi }{\mu (\alpha +\mu )}}\right) \end{array}$$

$$\begin{array}{c} {∴A}^{*}=\frac{\pi }{\mu }\left(\text{1}-\frac{\text{1}}{R_{\text{0}}}\right). \end{array}$$ (4)

The force of transmission to anxiety class is given by


$$\begin{array}{c} \vartheta =\;\beta S^{*}(t)A^{*}(t) \end{array}$$

$$\begin{array}{c} =\;\beta \left(\frac{\alpha +\mu }{\beta }\right)\left(\frac{\pi }{\mu }-\frac{\alpha +\mu }{\beta }\right) \end{array}$$

$$\begin{array}{c} =(\alpha +\mu )\left(\frac{\pi }{\mu }-\frac{\alpha +\mu }{\beta }\right) \end{array}$$

$$\begin{array}{c} =\frac{\pi (\alpha +\mu )}{\mu }\left(\text{1}-\frac{\mu (\alpha +\mu )}{\beta \pi }\right) \end{array}$$

$$\begin{array}{c} ∴\vartheta =\frac{\pi (\alpha +\mu )}{\mu }\left(\text{1}-\frac{\text{1}}{R_{\text{0}}}\right). \end{array}$$

Since the parameters π, α and μ are greater than zero, so ϑ is positive when $R_{\text{0}}>\text{1}$. If $R_{\text{0}}<\text{1}$, then at steady state the force of transmission ϑ  is negative. Hence, the model does not have any positive equilibrium. Thus the model has a unique positive anxiety prevailing equilibrium whenever $R_{\text{0}}>\text{1}$ and no positive equilibrium otherwise.

#### Local stability of anxiety prevailing equilibrium.

**Theorem 8:** The positive anxiety prevailing equilibrium of the system (1) is locally asymptotically stable on Ω when $R_{\text{0}}>\text{1}$.

Proof: The Jacobian matrix of the model (1) at $E^{*}$ is


$$\begin{array}{c} J\left(E^{*}\right)=[\begin{array}{cc} -\beta A^{*}-\;\mu & -\beta S^{*}+\alpha \\ \beta A^{*} & \beta S^{*}-(\mu +\alpha ) \end{array}]\;. \end{array}$$

Applying elementary row operation, we get,


$$\begin{array}{c} J\left(E^{*}\right)=[\begin{array}{cc} -\beta A^{*}-\;\mu & -\beta S^{*}+\alpha \\ \text{0} & \frac{\mu (\beta {(A^{*}-S}^{*})+\mu +\alpha )}{-\beta A^{*}-\;\mu } \end{array}]\;. \end{array}$$

To find the eigenvalues of the upper triangular matrix $J\left(E^{*}\right)$, we solve its characteristic equation $\left|J\left(E^{*}\right)-\lambda I\right|=\text{0}$; where I is a 2×2 identity matrix.


$$\begin{array}{c} \left|J\left(E^{*}\right)-\lambda I\right|=\left|\begin{array}{cc} -\beta A^{*}-\;\mu -\lambda_{\text{1}} & -\beta S^{*}+\alpha \\ \text{0} & \frac{\mu (\beta {(A^{*}-S}^{*})+\mu +\alpha )}{-\beta A^{*}-\;\mu }-\lambda_{\text{2}} \end{array}\right|=\text{0} \end{array}$$

$$\begin{array}{c} \Rightarrow \left(-\beta A^{*}-\;\mu -\lambda_{\text{1}}\right)\left(\frac{\mu (\beta {(A^{*}-S}^{*})+\mu +\alpha )}{-\beta A^{*}-\;\mu }-\lambda_{\text{2}}\right)=\text{0} \end{array}$$ (5)

$$\begin{array}{c} \;\begin{array}{c} ∴\lambda_{\text{1}}=-\beta A^{*}-\;\mu ,\\ \lambda_{\text{2}}=\frac{\mu (\beta {(A^{*}-S}^{*})+\mu +\alpha )}{-\beta A^{*}-\;\mu }. \end{array}\} \end{array}$$

Here, $\lambda_{\text{1}}<\text{0}$ and if $\lambda_{\text{2}}<\text{0}$, then


$$\begin{array}{c} \frac{\mu (\beta {(A^{*}-S}^{*})+\mu +\alpha )}{-\beta A^{*}-\;\mu }<\text{0} \end{array}$$

$$\begin{array}{c} ⟺\frac{\beta {(A^{*}-S}^{*})+\mu +\alpha }{-\beta A^{*}-\;\mu }<\text{0} \end{array}$$

$$\begin{array}{c} ⟺\beta {(A^{*}-S}^{*})+\mu +\alpha >\text{0} \end{array}$$

$$\begin{array}{c} ⟺\beta (\frac{\pi }{\mu }-\frac{\alpha +\mu }{\beta }-\frac{\alpha +\mu }{\beta })+\mu +\alpha >\text{0} \end{array}$$

$$\begin{array}{c} ⟺\frac{\beta \pi }{\mu }-\text{2}(\mu +\alpha )+\mu +\alpha >\text{0} \end{array}$$

$$\begin{array}{c} ⟺\frac{\beta \pi }{\mu }-(\mu +\alpha )>\text{0} \end{array}$$

$$\begin{array}{c} ⟺\frac{\beta \pi }{\mu (\mu +\alpha )}-\text{1}>\text{0} \end{array}$$

$$\begin{array}{c} ⟺R_{\text{0}}>\text{1}. \end{array}$$

Thus $\lambda_{\text{2}}<\text{0}$ if and only if $R_{\text{0}}>\text{1}$. It can be seen that all the eigenvalues of the characteristic polynomial (4) have negative real part if and only if $R_{\text{0}}>\text{1}$. Hence, it is proved that if $R_{\text{0}}>\text{1}$, then $E^{*}$ is locally asymptotically stable. Again, if $R_{\text{0}}<\text{1}$, then clearly $\lambda_{\text{2}}>\text{0}$. So, equation (4) has at least one positive eigenvalue which implies that $E^{*}$ is asymptotically unstable. Hence, the theorem is proved.

#### Global stability analysis of anxiety prevailing equilibrium.

**Theorem 9:** When $\;R_{\text{0}}>\text{1}$, the anxiety prevailing equilibrium $\;E^{*}\;$ is globally asymptotically stable on  Ω  if $\text{ sign}(S-S^{*})=\;\;\;\;/\;\text{sign}{(S}^{*}A^{*}-SA)\;$ and $\text{ sign}(S-S^{*})=\;\;\;\;/\;\text{sign}({A-A}^{*})$.

Proof: Let us consider the following Lyapunov function,


$$\begin{array}{c} U(S,A)=S-S^{*}-S^{*}\text{ln}\frac{S}{S^{*}}+A-A^{*}-A^{*}\text{ln}\frac{A}{A^{*}}. \end{array}$$

Here, U(S, A)>0 for $\left(S,A)=\;\;\;\;/\;(S^{*},\;A^{*}\right)$ and for $\left(S,\;A)=(S^{*},\;A^{*}\right)$,


$$\begin{array}{c} U(S,\;A)=\text{0}. \end{array}$$

Now, the derivative of U with respect to time is,


$$\begin{array}{c} \frac{dU}{dt}=\left(\text{1}-\frac{S^{*}}{S}\right)\frac{dS}{dt}+\left(\text{1}-\frac{A^{*}}{A}\right)\frac{dA}{dt} \end{array}$$

$$\begin{array}{c} \Rightarrow \frac{dU}{dt}=\left(\text{1}-\frac{S^{*}}{S}\right)(\pi -\beta SA+\alpha A-\mu S)+\left(\text{1}-\frac{A^{*}}{A}\right)(\beta SA-\;\alpha A-\;\mu A). \end{array}$$

At anxiety prevailing equilibrium $E^{*}$,


$$\begin{array}{c} \frac{dS^{*}}{dt}=\frac{dA^{*}}{dt}=\text{0}. \end{array}$$

Now,


$$\begin{array}{c} \frac{dS^{*}}{dt}=\text{0}\Rightarrow \pi -\beta S^{*}A^{*}+\alpha A^{*}-\mu S^{*}=\text{0} \end{array}$$

$$\begin{array}{c} ∴\;\pi =\beta S^{*}A^{*}-\alpha A^{*}+\mu S^{*}. \end{array}$$ (6a)

Again,


$$\begin{array}{c} \frac{dA^{*}}{dt}=\text{0}\Rightarrow \;\beta S^{*}A^{*}-\;\alpha A^{*}-\;\mu A^{*}=\text{0} \end{array}$$

$$\begin{array}{c} ∴\;\alpha +\mu =\;\beta S^{*}. \end{array}$$ (6b)

From (5a) and (5b) we get,


$$\begin{array}{c} \frac{dU}{dt}=\left(\text{1}-\frac{S^{*}}{S}\right)\left(\beta S^{*}A^{*}-\alpha A^{*}+\mu S^{*}-\beta SA+\alpha A-\mu S\right)+\left(\text{1}-\frac{A^{*}}{A}\right) \end{array}$$

$$\begin{array}{c} \left(\beta SA-\;\beta S^{*}A\right) \end{array}$$

$$\begin{array}{c} \Rightarrow \frac{dU}{dt}=\beta \left(\frac{{S-S}^{*}}{S}\right)(S^{*}A^{*}-SA)+\alpha \left(\frac{S-S^{*}}{S}\right)(A-A^{*})-\mu \frac{{\left(S-S^{*}\right)}^{\text{2}}}{S}+\beta A\left(\frac{{A-A}^{*}}{A}\right)(S-\;S^{*})\leq \text{0} \end{array}$$

where $\text{sign}(S-S^{*})=\;\;\;\;/\;\text{sign}{(S}^{*}A^{*}-SA)$ and $\text{sign}(S-S^{*})=\;\;\;\;/\;\text{sign}({A-A}^{*})$. Hence by Lyapunov stability theorem, it is verified that $E^{*}$ is globally asymptotically stable on Ω for the condition mentioned above.

### Bifurcation analysis

#### Existence of transcritical bifurcation.

We have shown that the anxiety free equilibrium is locally asymptotically stable for $R_{\text{0}}<\text{1}$ and unstable for $R_{\text{0}}>\text{1}$. The analysis becomes ineffective for $R_{\text{0}}=\text{1}$ as one of the eigenvalues of the Jacobian matrix is zero in this case.

Now, $R_{\text{0}}=\text{1}$ is equivalent to


$$\begin{array}{c} \beta =\tilde{\beta }=\frac{\mu (\alpha +\mu )}{\pi }. \end{array}$$

In this case, we verify the transversality conditions of Sotomayor’s theorem [42] to investigate the phenomenon of transcritical bifurcation at anxiety free equilibrium $E^{\text{0}}$. A transcritical bifurcation is a type of local bifurcation where two equilibrium states meet and then interchange their stability as a control parameter passes through a critical point. At the bifurcation point, both equilibria exist for parameter values on either side, but their stability properties are interchanged as the parameter crosses the critical value. [43,44]

Let, $\vec{g}={\left(g_{\text{1}},\;g_{\text{2}}\right)}^{T}$; where,


$$\begin{array}{c} g_{\text{1}}=\pi -\beta SA+\alpha A-\mu S, \end{array}$$

$$\begin{array}{c} g_{\text{2}}=\beta SA-(\alpha +\mu )A. \end{array}$$

Let, $V={\left(v_{\text{1}},\;v_{\text{2}}\right)}^{T}$ and $W={\left(w_{\text{1}},\;w_{\text{2}}\right)}^{T}$ be the eigenvectors of ${J\left(E^{\text{0}}\right)}_{\beta =\tilde{\beta }}$ and ${{(J\left(E^{\text{0}}\right)}_{\beta =\tilde{\beta }})}^{T}$ respectively corresponding to the zero eigenvalue,

where,


$$\begin{array}{c} v_{\text{1}}=-\text{1},\;v_{\text{2}}=\text{1}.\; \end{array}$$

$$\begin{array}{c} w_{\text{1}}=\text{0},\;w_{\text{2}}=\text{1}. \end{array}$$

Now,


$$\begin{array}{c} W^{T}g_{\beta }\left(E^{\text{0}};\tilde{\beta }\right)=\text{0} \end{array}$$

$$\begin{array}{c} W^{T}\left(Dg_{\beta }\left(E^{\text{0}};\tilde{\beta }\right)V\right)=\frac{\pi }{\mu }=\;\;\;\;/\;\text{0} \end{array}$$

$$\begin{array}{c} W^{T}\left(D^{\text{2}}g\left(E^{\text{0}};\tilde{\beta }\right)(V,\;V)\right)=-\frac{\text{2}\mu (\alpha +\mu )}{\pi }=\;\;\;\;/\;\text{0} \end{array}$$

Therefore, all the requirements in Sotomayor’s theorem [42] are satisfied. Hence, a transcritical bifurcation occurs at $E^{\text{0}}$ when the parameter β crosses its critical value


$$\begin{array}{c} \beta =\tilde{\beta }=\frac{\mu (\alpha +\mu )}{\pi }. \end{array}$$

**Fig 2:**
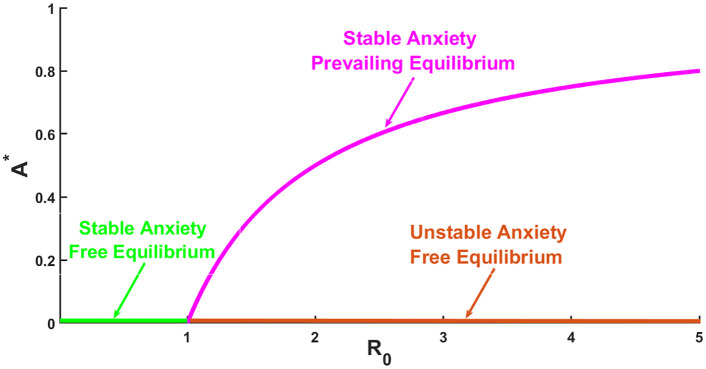
Transcritical Bifurcation Diagram (π=0.0001, μ=0.0001)).


Fig 2 shows the transcritical bifurcation diagram of the model. To generate the bifurcation diagram, we use equation (4) in Theorem 7, which is:

$$\begin{array}{c} A^{*}=\frac{\pi }{\mu }\left(\text{1}-\frac{\text{1}}{R_{\text{0}}}\right). \end{array}$$

In Fig 2, $A^{*}$ is the dependent variable and $R_{\text{0}}$ is the independent variable (bifurcation parameter). So, the bifurcation parameter $R_{\text{0}}$ is varied systematically $\left(\text{0}\;\leq \;R_{\text{0}}\;\leq \;\text{5}\right)$ while all other parameters (π  =  0.0001, µ  =  0.0001) remain fixed in order to generate changes in equilibrium point $A^{*}$ and its stability.

### Sensitivity analysis

**Fig 3:**
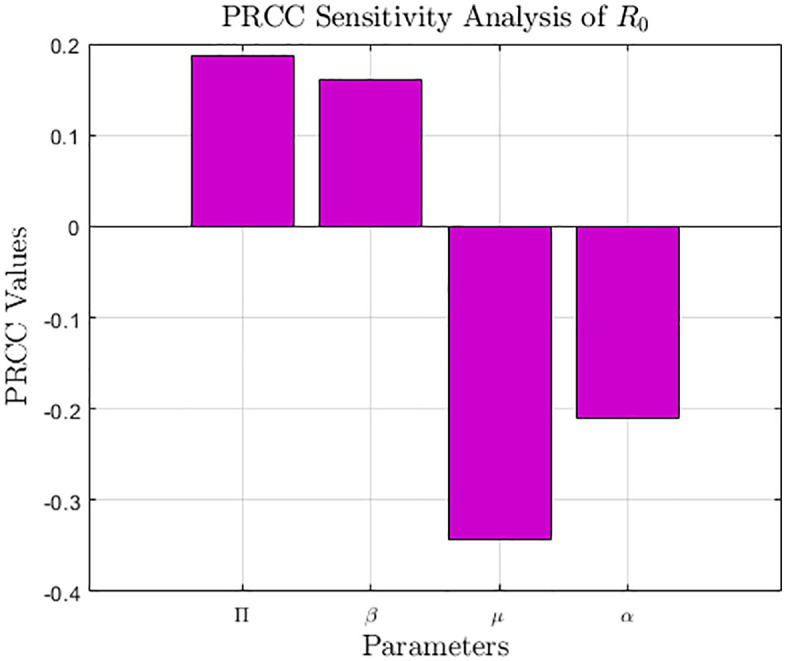
PRCC Sensitivity Analysis of R_0_.

We employ Partial Rank Correlation Coefficient (PRCC) method to determine the most dominant parameters of the SAS model. PRCC is a global sensitivity analysis technique used to evaluate the relationship between the model output and the model parameters [45]. PRCC value ranges from −1 –1 where a negative value represents an inverse relation between the output and the corresponding input parameter, whereas a positive value indicates a proportional correlation between them [45]. Fig 3 shows the PRCC value of R_0_ with respect to the four model parameters. The results indicate that the university entrance rate (π) and the social influence rate (β) both have a positive impact on R_0_. This means that an increase in the number of incoming students or stronger social transmission of anxiety raises the risk of math anxiety spreading. In contrast, the rate of recovery from anxiety (α) and the rate of exit from the system (μ) exhibit a negative relationship with R_0_. This suggest that interventions helping students overcome anxiety and natural turnover (graduation or dropout) reduce the likelihood of math anxiety persisting in the population.

### Numerical solutions

**Fig 4:**
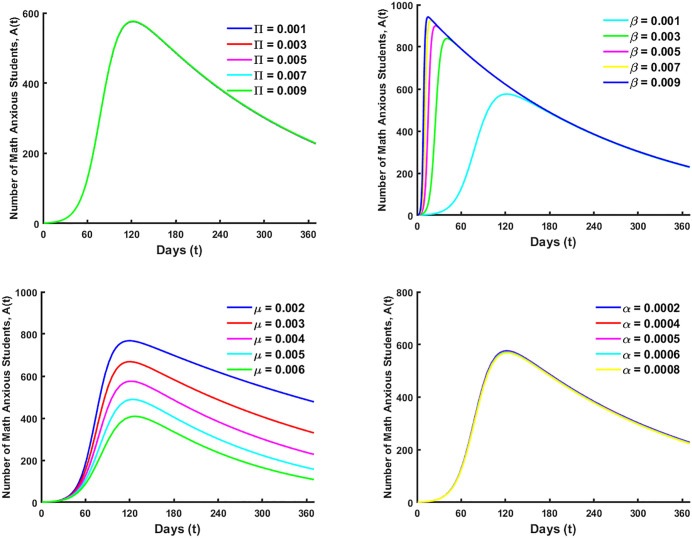
Variation in the population of Class A due to fluctuations in the parameters. (a), (b) β, (c) μ, and (d) α.

Fig 4 suggests that the population size of students with mathematics anxiety (class A) is highly sensitive to changes in the social transmission rate (β) and the exit rate (μ), while it remains relatively stable with small fluctuations in the university entrance rate (π) and the recovery rate (α). This indicates that peer influence and student retention play a more critical role in shaping the anxiety dynamics than the rate at which students enter the university or recover from anxiety. In practical terms, even a slight increase in social pressure or exposure to anxious peers can significantly raise anxiety levels among students. Similarly, changes in the rate of graduation or dropout affect how long anxious students remain in the system, thereby influencing the total size of the anxious population. On the other hand, small improvements in support programs (α) or variations in incoming student numbers (π) have a limited short-term impact, suggesting that targeting social dynamics and retention policies may be more effective in managing and reducing math anxiety within the student body.

**Fig 5:**
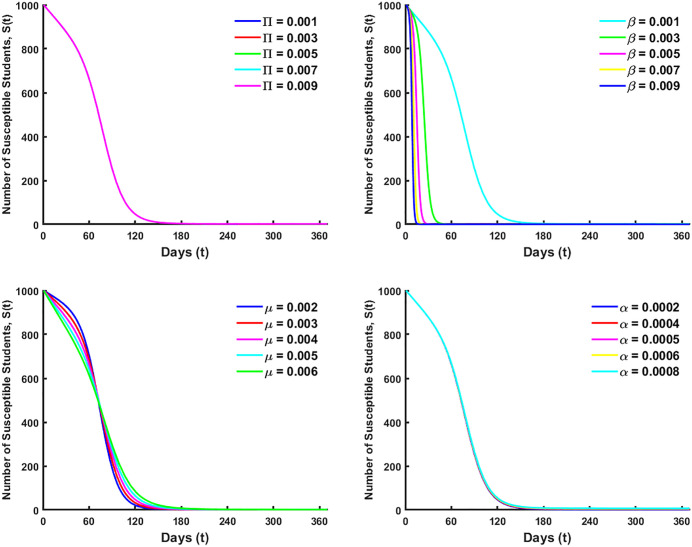
Variation in the population of class S due to fluctuations in the parameters. (a) π, (b) β, (c) μ, and (d) α.

Fig 5 indicates that the population size of susceptible students (class S)—those without mathematics anxiety—is significantly influenced by small changes in the social transmission rate (β) and the exit rate (μ). In contrast, minor variations in the university entrance rate (π) and the recovery rate from anxiety (α) have little impact on the susceptible population. This suggests that the rate at which anxiety spreads through social interactions (β) and the rate at which students leave the system (μ) are key drivers of changes in the non-anxious student group. An increase in β reduces the susceptible population as more students transition to the anxious class, while variations in μ affect the overall population dynamics by altering the duration students remain in the system. On the other hand, modest changes in how many students enter the university or recover from anxiety do not substantially alter the size of the susceptible group.

### Controlling mathematics anxiety

**Fig 6:**
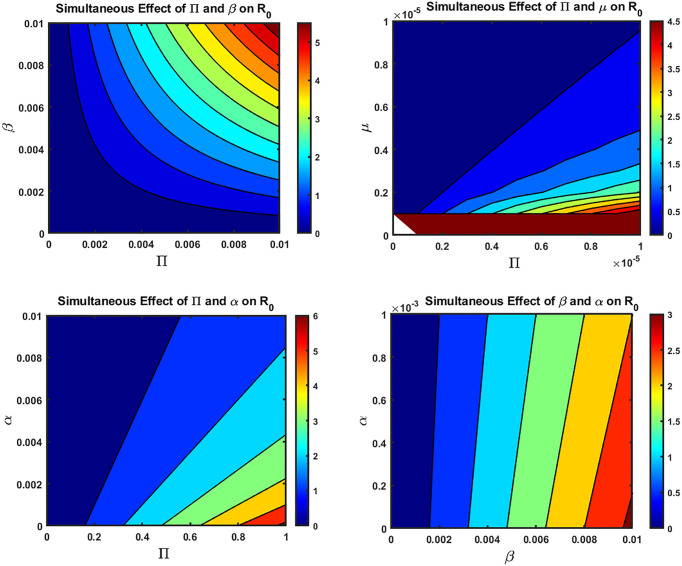
Simultaneous effect of the parameters (a) β and Π, (b) μ and Π, (c) α and Π, and (d) α and β on R_0_.

The contour plots in Fig 6 provide valuable insights into how different combinations of model parameters influence R_0_, which serves as a threshold indicator for the persistence or decline of mathematics anxiety within the student population. To effectively control and reduce the spread of math anxiety, it is essential to maintain R_0_  <  1. In particular, Fig 6(a) shows that simultaneously increasing the social transmission rate (β) and the university entrance rate (π) causes a steady rise in R_0_, suggesting that greater peer influence and larger incoming student cohorts can accelerate the spread of anxiety. Similarly, Fig 6(b) demonstrates that increasing π while decreasing the exit rate (μ) also raises R_0_, indicating that a longer stay in the system combined with a high influx of students can intensify the anxiety burden. In contrast, Fig 6(c) reveals that reducing π and enhancing the recovery rate (α) representing effective interventions or coping mechanisms that can lead to a marked decrease in R_0_, thus curbing the spread of anxiety. Notably, Fig 6(d) shows that keeping β below a critical threshold (β  <  0.002) ensures that R_0_ stays below one, regardless of the recovery rate, emphasizing the pivotal role of minimizing social transmission in controlling math anxiety.

## Conclusion

In this article, we investigated the classical SIS model adapted to capture the dynamics of math anxiety. The model admits two equilibrium points: the anxiety-free equilibrium and the anxiety-prevailing equilibrium. Theoretical analysis established that the anxiety-free equilibrium is both locally and globally asymptotically stable when $R_{\text{0}}<\text{1}$, whereas the anxiety-prevailing equilibrium is both locally and globally asymptotically stable for $R_{\text{0}}>\text{1}$. Furthermore, the system undergoes a transcritical bifurcation as the basic reproduction number passes through the threshold value.

The PRCC analysis revealed the most influential parameters affecting $R_{\text{0}}$. Specifically, an increase in the parameters π and β elevates the reproduction number, while higher values of μ and α reduce it. Sensitivity analysis further indicated that small fluctuations in β and μ substantially alter the population size of the anxious class A. In particular, an increment in β amplifies the size of the anxious population, whereas an increment in μ suppresses it.

These insights not only deepen the theoretical understanding of math anxiety but also provide practical guidance for educators and policymakers. Specifically, reducing exposure to anxiety- inducing practices and strengthening recovery through counseling, stress management, and supportive teaching can significantly curb its prevalence. Cognitive Behavioral Therapy (CBT), mindfulness training, cooperative learning etc. play significant role in this regard. Future extensions of this work may include stochastic effects, network structures of social influence, or time-dependent interventions, thereby offering a more realistic and robust framework for combating math anxiety. In addition, this framework can be extended by incorporating additional factors such as memory effects, heterogeneity among students, or external interventions such as awareness campaigns, which could yield richer insights into managing and mitigating math anxiety at both individual and societal levels.

### Declarations

**Use of Artificial Intelligence Tools:**

Generative artificial intelligence (AI) tools, specifically ChatGPT (GPT-3.5 model), were used in this study to assist with language refinement, grammar checking, and improving the clarity and flow of the manuscript. The AI tool was not used to generate or analyze data, interpret results, or draw scientific conclusions.
